# Leveraging the Power of High Performance Computing for Next Generation Sequencing Data Analysis: Tricks and Twists from a High Throughput Exome Workflow

**DOI:** 10.1371/journal.pone.0126321

**Published:** 2015-05-05

**Authors:** Amit Kawalia, Susanne Motameny, Stephan Wonczak, Holger Thiele, Lech Nieroda, Kamel Jabbari, Stefan Borowski, Vishal Sinha, Wilfried Gunia, Ulrich Lang, Viktor Achter, Peter Nürnberg

**Affiliations:** 1 Cologne Center for Genomics, University of Cologne, Cologne, Germany; 2 Regional Computing Center Cologne, University of Cologne, Cologne, Germany; CNRS UMR7622 & University Paris 6 Pierre-et-Marie-Curie, FRANCE

## Abstract

Next generation sequencing (NGS) has been a great success and is now a standard method of research in the life sciences. With this technology, dozens of whole genomes or hundreds of exomes can be sequenced in rather short time, producing huge amounts of data. Complex bioinformatics analyses are required to turn these data into scientific findings. In order to run these analyses fast, automated workflows implemented on high performance computers are state of the art. While providing sufficient compute power and storage to meet the NGS data challenge, high performance computing (HPC) systems require special care when utilized for high throughput processing. This is especially true if the HPC system is shared by different users. Here, stability, robustness and maintainability are as important for automated workflows as speed and throughput. To achieve all of these aims, dedicated solutions have to be developed. In this paper, we present the tricks and twists that we utilized in the implementation of our exome data processing workflow. It may serve as a guideline for other high throughput data analysis projects using a similar infrastructure. The code implementing our solutions is provided in the supporting information files.

## Introduction

Next generation sequencing has been a great success and is increasingly used as a research method in the life sciences. It enables researchers to decipher genomes with unprecedented resolution, study even subtle changes in gene expression, and identify disease-causing mutations in high throughput fashion. These are just a few examples where NGS has pushed ahead the frontiers of research, but it comes with a downside: Complex bioinformatics analyses have to be performed to turn the NGS data into scientific findings. When large data sets from whole genome sequencing or thousands of exomes have to be analyzed, more storage and compute power is required than most conventional hardware can provide. Therefore, automated NGS data analysis workflows implemented on high performance computers have become state of the art. For example, the MegaSeq [1] and HugeSeq [2] workflows implement whole genome sequencing data analysis on HPC systems. These workflows use the MapReduce [3] paradigm by repeatedly splitting the data into chunks that are processed in parallel followed by merging of the results. This strategy is the main measure to speed up the data analysis and we also use it in our workflow. NGSANE [4] is a framework for NGS data analysis implemented in BASH [5]. The implementation is rather similar to ours but differs in the selection of tools and details of the implementation. WEP [6] and SIMPLEX [7] provide integrated workflows for exome analysis either as a web-interface (WEP) or a VirtualBox and Cloud Service (SIMPLEX), enabling users with little bioinformatics knowledge to analyze their data on remote HPC systems. In [8], a general approach to NGS data management is outlined that comprises all steps from sample tracking in the wet-lab over data analysis to presentation of the final results. Here, HPC is used in the data analysis step. While all of these publications report the use of HPC to accelerate NGS data analysis, few of them describe the specific details of the implementation. In [1], [2], and [4], only the parallelization strategies to speed up the analysis are discussed in detail. [6] and [7] do not provide any details about the HPC implementation and [8] only briefly mentions the automatic generation of jobs for the HPC system. When we developed our automated workflow for exome analysis, we found that stability, robustness and maintainability are as important to consider as speed. We here present all the dedicated solutions that we implemented to achieve not only fast runtimes of the analysis but to run the workflow robustly in a multiuser HPC environment taking care to not destabilize the system.

Like the workflow presented in [4], our workflow is implemented in BASH and can therefore be used in any Linux environment without further dependencies. Although many of its parts are of the MapReduce kind, we did not consider Hadoop [9] for the implementation because it is not widely used in our field of research and is not well suited for our HPC system. Our HPC system is a shared cluster that is used by researchers from different sciences. Therefore, its architecture is rather generic and balanced between number crunching and data analysis. We believe that such kind of resources are available to many researchers in the scientific community who face a growing demand for computational power but cannot host an HPC system themselves. In this situation, it is extremely important to include measures for system stability and robustness in the implementation of automated workflows. HPC systems have a complex architecture with many components that have to work smoothly together (see Paragraph “The HPC Environment” for a detailed description of our HPC system). As the failure probabilities of the single components sum up, the failure of a single component is a rather common event compared to a commodity server. Also, numerous bottlenecks can arise, for example, in job scheduling or accessing of the parallel filesystem, which are unique to HPC systems. Therefore, they are much less tolerant than common servers and can easily be destabilized when not paying attention. In a multiuser environment, all users will be affected and hampered in their research if your workflow causes instabilities. On the other hand, you cannot control what other users do on the system, so your workflow should be able to react to the bottlenecks they may introduce. Therefore, we took special measures during the development of our workflow implementation to overcome bottlenecks and avoid system instabilities. Moreover, it was crucial for us that the workflow runs without user interaction to the most possible extent. When large sets of samples have to be analyzed, we cannot afford to debug every error manually. The HPC environment introduces new classes of errors that originate from the system’s resource management, e.g., computations fail because not enough memory or walltime (the wall-clock time (in contrast to CPU-time) the job is allowed to run on the HPC system) was requested. Our implementation can handle these errors automatically so manual debugging is restricted to the errors that are thrown by the computation itself.

Finally, next generation sequencing is very versatile. There are a lot of different applications that require different kinds of analyses with new ones arising as the technological development advances. Even greater is the wealth of available tools for NGS data analysis with almost daily new releases. In this rapidly evolving field of research, it is impossible to design a workflow once that will then stay in production for several years. Instead, new tools have to be added, outdated ones have to be dropped and different applications have to be integrated. Therefore, maintainability of the code was also an important factor that we considered during the development.

Our achieved solution is running very successfully. With the current implementation we reach a throughput of 290 exomes per week with an average runtime of 21 hours per exome. This throughput and speed matches well with our current sequencing capacities and is considerable for a shared commodity HPC cluster.

In this paper, we present the dedicated solutions we used to achieve high throughput, stability, robustness, and maintainability of our workflow. Although developed for NGS data analysis, they are not restricted to this type of application but can be utilized for any high throughput data processing project on a similar HPC infrastructure.

## Workflow Overview

The focus of this paper is not on our exome analysis workflow itself. We rather present the dedicated solutions we found during its development to run it in high throughput fashion in a multiuser HPC environment. Nevertheless, we first give a short overview of our workflow and infrastructure in order to provide the context for the following discussion.

### Workflow and Components

**Fig 1:**
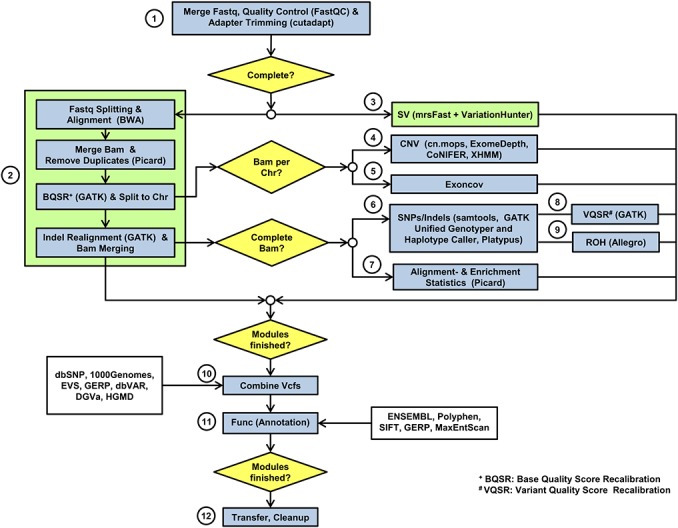
Exome Analysis Workflow. Checkpoints where a workflow abortion can be triggered are colored in yellow, subpipelines are colored in green.

**Fig 2:**
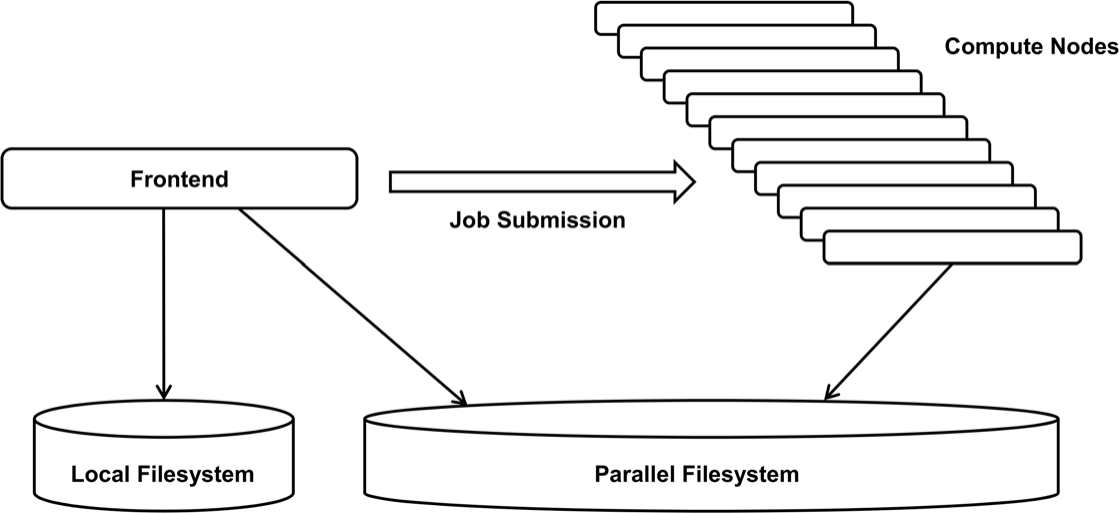
Schematic of the HPC Working Environment.

**Fig 3:**
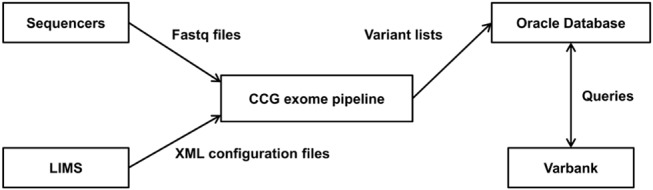
Workflow Context.

**Fig 4:**
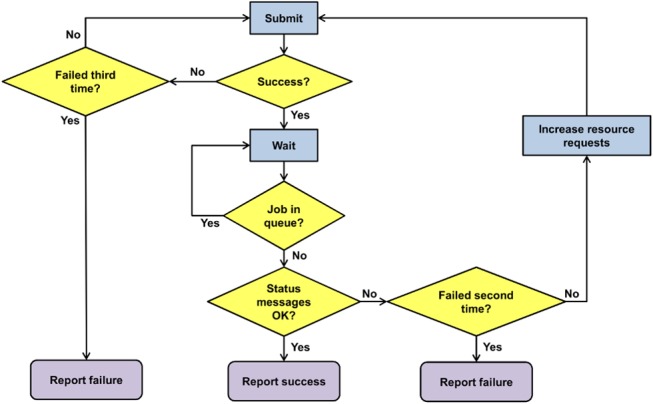
Flowchart of the Job Submission Function.

**Fig 5:**
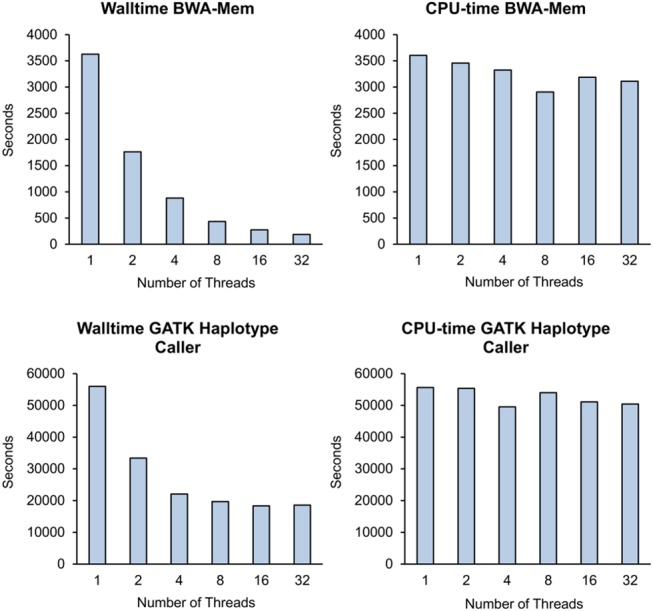
Performance of Multi-Threading. CPU-time and walltime usage of BWA-Mem and GATK HaplotypeCaller with different number of threads.

Our exome analysis workflow is a collection of open source third party tools and self-developed software that are stitched together into a pipeline via BASH scripts. While other published exome analysis workflows are limited to SNP/indel calling [6–8], our implementation includes both copynumber variant calling based on depth of coverage and structural variant calling based on discordant mate pairs. It has proven its utility in multiple research projects and successfully uncovered the genetic background of various diseases (see, e.g., [10–13]). A full exome analysis for an individual sample runs as follows (Fig 1): The fastq files are first prepared for analysis. This includes merging of all available fastq files for the sample (self-written script), a quality check, and adapter trimming using FastQC [14] and cutadapt [15] (Fig 1, (1)). The prepared fastq files go through structural variant calling on the one hand and alignment for SNP/indel calling on the other hand. Both of these tasks first split the fastq files into chunks and align them to the reference genome. The resulting alignment files are merged and further processed. For structural variant calling, a strict alignment allowing only perfect matches is performed using mrsFast [16] and then structural variants are detected based on discordant mate pair signatures by VariationHunter [17] (Fig 1, (3)). The alignment procedure for SNP/indel calling uses BWA [18] and is much more sensitive allowing for gaps and mismatches (Fig 1, (2)). Here, after merging of the bam files from the independently aligned chunks by samtools [19], PCR duplicates are removed next using Picard [20]. After this, basecalling quality scores are recalibrated using GATK [21] and the bam file is split into 25 bamfiles by samtools, one for each chromosome and the mitochondrion. This is done to speed up the following indel realignment, which is run chromosome-wise using GATK. The chromosomal bam files are also used to call copynumber variants by four different callers (CoNIFER [22], XHMM [23], cn.nops [24], and ExomeDepth [25], Fig 1, (4)) and compute statistics about exon coverage throughout the genome (self-written script, Fig 1, (5)). After indel realignment (GATK), the chromosomal bam files are merged again (samtools) and the SNP/indel calling is performed using samtools mpileup, GATK UnifiedGenotyper and GATK HaplotypeCaller, and Platypus [26] (Fig 1, (6)). Also, alignment- and enrichment statistics are computed on the complete bam file using Picard (Fig 1, (7)). Following SNP/indel calling, variant quality score recalibration is performed on the variant lists by GATK (Fig 1, (8)) and regions of homozygosity (ROH) are detected using Allegro [27] (Fig 1, (9)). After completion of all these analysis steps, the results are collected and combined into one vcf file (self-written script, Fig 1, (10)). Several databases are parsed to annotate known variants during this step as well (dbSNP [28], 1000 Genomes Project [29], Exome Variant Server (EVS http://evs.gs.washington.edu/EVS/), dbVAR and DGVa [30], GERP [31], ENSEMBL [32], and the commercial HGMD professional database [33]). Next, a functional assessment of the variants is performed by combining various third party tools (POLYPHEN [34], SIFT [35]) and algorithms developed inhouse in a self-written script (Fig 1, (11)). The splice site analysis is based on [36] and also performed in this step. The completely annotated variant list is prepared for transfer to its final destination and the intermediate results are deleted (self-written scripts, Fig 1, (12)).

The workflow contains several checkpoints, which are depicted as diamonds in Fig 1. If the condition at any checkpoint is violated, the analysis is aborted. In this case, the workflow waits for all modules that have already been started to finish and then exits with an error message.

In most of the cases, we analyze NGS exome data sample-wise in our implementation, i.e., a full analysis is performed for every sample individually. One exception to this is the detection of denovo mutations in trios, twins, or tumor-normal pairs. For this purpose, we use deNovoGear [37] that is run in addition to the above described analysis steps for exome data from the affected child of a trio, the affected twin of a sibling pair or the tumor of a tumor-normal pair.

### The HPC Environment

The workflow is implemented on the HPC clusters CHEOPS and SuGI of the Regional Computing Center Cologne (RRZK). These clusters are central resources of the University of Cologne and used by a multitude of researchers from different sciences. CHEOPS has 841 compute nodes with a total of 9712 cores, 35.5 TB RAM, and 500 TB Lustre parallel file storage. With a peak performance of 100 Teraflop/s and linpack performance of 85.0 Teraflop/s, it occupied rank 90 among the 500 world’s fastest supercomputers in 2010. SuGI has 32 compute nodes with a total of 256 cores, 1 TB RAM and 5 TB Panasas parallel file storage. It reaches a peak performance of 2 Teraflop/s. Both clusters run a Linux operating system. Working on an HPC cluster differs from working on a standard desktop computer or server in a fundamental way: computations cannot be run directly but have to be submitted as jobs. Job submission takes place on dedicated nodes, the frontend nodes, of the cluster. In addition to job submission, the frontend nodes are also used for login and data transfer from and to the parallel filesystem. All computation is encapsulated in jobs that are submitted to the compute nodes via a batch system. In our case, the batch system is SLURM [38] on CHEOPS and TORQUE [39]/Maui on SuGI. When submitting a job, resources like number of cores, required memory, and walltime, are requested via options of the submission command. Submitted jobs are then assigned to appropriate compute nodes by the batch system’s scheduler. A schematic of the cluster structure is shown in Fig 2.

Following this way of working on a cluster, our exome workflow is coded as a collection of BASH scripts which are divided into one masterscript and several jobscripts. The sole purpose of the masterscript is the organization of the workflow and submission of the jobscripts. These jobscripts then do the actual computation.

### Workflow Context

The workflow is configured for each sample individually by passing a configuration file in XML (Extensible Markup Language [40]) format to the masterscript. XML provides a standard format for hierarchically structured textual data and many tools support reading and writing of XML files. The configuration file is generated from our LIMS (Laboratory Information Management System, a database that keeps track of all samples and their processing in the wet-lab) and lists all necessary information relevant to the analysis.

As result, the workflow produces a list of annotated variants which is uploaded to an Oracle database. This database can be queried by the researchers via a webinterface called “varbank” (https://varbank.ccg.uni-koeln.de). The varbank webinterface also provides access and download options for the fastq-, bam-, and vcf files as well as annotated variant tables and coverage statistics. A description of varbank is out of the scope and will thus be published separately. Fig 3 shows the infrastructure surrounding the exome workflow.

## Technical Details

### Workflow Masterscript

The masterscript controls the analysis for one data set (in our case for one exome). Its sole purpose is the organization of the workflow and submission of the jobscripts that do the actual computation. Several instances of the masterscript are run in parallel to achieve high throughput. They are started automatically by the cron deamon on the frontend node when new data is available for processing.

#### Job submission

Core to the masterscript is a job submission function. This function submits a jobscript for execution on the compute nodes, monitors its status, and checks whether it finishes successfully. Fig 4 shows a flowchart of the job submission function.

The job submission function makes up to three attempts to submit a job. If all three attempts fail (e.g., because the batch system is unavailable), an error is reported. After successful submission, it waits for the job to finish. If the job fails, it is automatically resubmitted with increased resource requests (memory and walltime). If a resubmitted job fails again, an error is reported. The job submission function currently supports two batch systems: SLURM, which is running on CHEOPS, and TORQUE/Maui, which is running on SuGI. It provides a general job submission syntax to the masterscript and translates it into the respective batch system’s job submission commands.

The code of the job submission function is contained in the S1 Supporting Information.

#### Workflow organization

The masterscript uses two modes to submit jobs. Either, it calls the job submission function directly, causing the masterscript to wait until the submitted job is completed, or it starts a child process in the background that calls the job submission function. The first strategy is used when jobs are logically dependent on each other, so that one job has to be completed before the next one can start. The second strategy is used when jobs are independent from another and can run in parallel. For example, the initial preparation of the fastq input files including quality check and adapter trimming is submitted by the masterscript directly, because all other jobs depend on it. After this job has completed, the alignment jobs for SNP/indel calling and SV detection are run in parallel via child processes, because they are independent from another. This way, the masterscript organizes the workflow as a series of job submissions (either directly or via child processes) and checkpoints. At every checkpoint, the masterscript waits for the submitted jobs and started child processes to finish. This is done by checking the queue (every job is monitored separately by the submission function) and the process table on the frontend node. Once all started computations have finished, the masterscript checks whether there were any errors or failures. If this is the case, the workflow is aborted with an error message, otherwise it moves on to the next round of job submissions.

#### Job tracking and clean exit

The masterscript keeps track of all directly submitted jobs via the job ids that are assigned to them by the batch system. These job ids are all stored in a dedicated variable of the masterscript. Also, all process ids of started child processes are tracked in a dedicated variable. When the masterscript is interrupted or aborted for any reason, all submitted jobs are deleted and all started child processes are killed automatically by means of a special cleanup function. The cleanup function is triggered by the “trap” statement and executed whenever the masterscript exits. Also, the child processes have a cleanup function that deletes their submitted jobs. This way, a clean exit is ensured if the workflow is interrupted (S3 Supporting Information).

#### Modularity

The masterscript has a modular structure, summarizing tools that perform similar tasks into one module. The modules which are to be executed can be selected by a command line option of the masterscript (S5 Supporting Information). This saves much time in case of failures because only failed modules have to be rerun. The modularity is also exploited during automatic operation of the workflow where we select the modules to be run according to the type of sample. For example, we skip the SV detection module for single read samples as mate-pair information is necessary for this type of analysis.

#### Job Scripts

The job scripts are BASH scripts that execute the software for processing the data. They are also used to prepare the data for analysis. Every jobscript writes a status message to the local file system on the frontend node when it exits. If the job is aborted by the batch system (e.g., because it exceeded the requested walltime or memory limits), the status message will not be written. This way, execution errors can be distinguished from aborted jobs. The status messages are checked by the masterscript when it reaches a checkpoint. When they indicate an error or when some of them are missing, the masterscript aborts the workflow and reports an error.

## Design Principles

Four design principles guided the development of the workflow code:

1. Speed: Timely and fast data analysis
2. Stability: Careful handling of the HPC environment
3. Robustness: Automatic reaction to processing problems
4. Maintainability: Easy operation and extension

In this section we present how our code meets each of these principles.

### Speed

#### Parallelization by Jobarrays

In order to make best use of the compute power of the clusters, many tasks of the workflow are parallelized. This is done by splitting the data into chunks, processing each chunk separately, and merging the results from the chunks to the final result. This principle of “parallelization by chunks” works for all tasks where every data record is processed independently of other data records. We do this for BWA and mrsFast alignments by splitting the fastq files. In addition to splitting the fastq files, we also use multiple threading in BWA which further speeds up the analysis (see also next subsection). However, we cannot use more than 8 threads in any job of the workflow because SuGI only has 8 core nodes. Therefore, a combination of splitting into chunks and multiple threading works best for us.

For GATK indel realignment, VariationHunter and deNovoGear, we split the bam file by chromosome (similar to [1] and [2]) and run the analysis on the chromosomes in parallel.

Technically, we implemented parallelization by chunks via jobarrays. A jobarray is a collection of jobs (called tasks) that runs the same computation for different input files. The advantage of using a jobarray is that all jobs are submitted at once and can be addressed by a single jobid. This greatly facilitates the submission procedure and job monitoring. Array size and runtime of the individual array tasks are controlled by the chunk size, which has to be chosen carefully. If it is too high, processing times of the individual tasks will be long and the parallelization capacities of the cluster are not well exploited. On the other hand, if it’s too low, processing times of a single task will be fast but the system will be flooded with jobs and waiting times in the queue will be increased. The sweet spot where the workflow runs most efficiently has to be found by trial and it can shift depending on the number of exomes run in parallel and the overall load on the cluster. In our implementation, we go by the rule that a single array task should run at least one hour. Also, we are trying to balance the runtimes of all tasks within a jobarray. If one task runs much longer than all the other ones, all jobs depending on the jobarray have to wait for it to finish and parallelization power is not fully exploited. Runtimes of array tasks can differ extremely if the chunks are of different size, e.g., when we split by chromosome. That is why we distribute the 24 chromosomes in this case across 14 tasks of a jobarray:

- chr 1 to 7: each chr in a single task
- chr 8 to 17: two chromosomes per task
- chr 18,19 and 20 processed in one task
- chr 21, 22, X and Y processed in one task

This strategy is a rule of thumb that works satisfactorily. However, the runtimes of the individual array tasks are also determined by other factors than chromosome size (e.g., coverage or mutation load), so there is no combination of chromosomes that is optimal for all data sets.

#### Parallelization by Threads

Some of the tools we use in the workflow offer a multi-threading option. We use this option to speed up the runtime of BWA, GATK, and Picard. Even though multi-threading is available for all of these tools, they behave quite differently when looking at the runtime. Fig 5 shows CPU time and walltime used by BWA and GATK HaplotypeCaller when run with different numbers of threads (run on a compute node with 4 Nehalem EX Octo-Core Processors, Xeon X7560, 2.27GHz).

The result shows that in general, walltime decreases with higher number of threads. But while BWA shows the expected acceleration, GATK HaplotypeCaller’s walltime does not decrease significantly beyond 4 threads. We are still investigating the reason behind this behavior but believe that this is due to the Java implementation of GATK. We have observed that Java applications produce an overhead of I/O operations that limit their acceleration capacity on the HPC system.

Another drawback we observed in GATK is that when run with more than one thread, the VariantRecalibrator module writes many intermediate output files (so called vcf stubs). The number of such files per exome analysis can easily reach 100000. As most filesystems have a quota on the number of files, this behavior limits the number of analyses that can run in parallel. We removed this bottleneck from our workflow by running the GATK VariantRecalibrator with only one thread. With this setting, the runtime is still fast and no intermediate files are produced.

#### Scalability

In HPC, scalability is an important measure for the performance of parallelized applications. There are two flavors, strong and weak scalability. Strong scalability measures how the runtime of an application changes when the number of cores is increased for a fixed problem size (we measured the strong scalability of BWA and GATK HaplotypeCaller in the above paragraph). Weak scalability measures how the runtime changes when the amount of work directed to a single core remains fixed but more cores are used due to a larger problem size. The “parallelization by chunks” strategy we use for BWA and mrsFast makes these parts of our workflow scale nearly perfect in the sense of weak scalability. The reason is that we split the data into chunks of equal size and processing of every chunk takes the same amount of time. Therefore, these parts of the workflow take almost the same amount of time for data sets of different sizes (not including queuing time). However, the scalability of the workflow as a whole is not easily assessed, because most parts work with a fixed number of cores. Also, scalability is inherently application dependent while we focus here on the general solutions to make a workflow fast, safe and robust in a shared HPC environment. These solutions are independent of the application and do not affect scaling.

#### Resource Usage Optimization

To achieve a satisfactory performance, a single analysis should run fast and the throughput should be high when running several analyses in parallel. To reach this, the resource requirements of the jobs in terms of number of cores and memory have to be optimized. This is a tedious task that requires many trial and error steps during the development of an automated workflow, but once these parameters are fixed, manual intervention is rarely necessary. There are a few guidelines that help to optimize the resource requests in order to achieve a high throughput workflow:

In general, large jobs requesting many cores and much memory at submission time have longer queuing time than small jobs that need only few cores and little memory. Also, the memory that is requested for a job at submission time is exclusively granted to that job, meaning that it is blocked for other jobs even if it is not used. If there are many jobs requesting more memory than they actually need, the cluster is partly idle while jobs wait in the queue. Therefore, resource requests should be as close to the true resource requirements of a job as possible.

In the case of jobarrays, the resources requested at submission time are valid for every jobarray task. But for some tools, it is very difficult to balance the memory usage of the jobarray tasks. In this case, a few tasks will take much more memory than the majority of the tasks. It is then beneficial to submit the jobarray first with a low memory request so that the majority of tasks can run in parallel with short queuing times and good exploitation of resources. Then, the failed tasks can be resubmitted with higher memory requests. This strategy prevents unnecessary blocking of resources and more analyses can run in parallel.

In addition to providing realistic estimates of the required resources, one should also consider the architecture of the cluster when optimizing resource requests. Our cluster CHEOPS provides nodes with 8, 12, and 32 cores. So when we run jobs with 1, 2, or 4 cores in the workflow, we can pack all such nodes with jobs. With 5 core jobs, we would end up with 3 or 2 cores that cannot be used for another job of the workflow, depending on the node type. So it is beneficial to use a number of cores that is a divisor of the number of cores available on the nodes whenever possible.

Similarly, the memory requests for the jobs should be chosen according to the available hardware. On CHEOPS, the majority of nodes have 24 or 48 GB RAM and a few have 96 or 512 GB RAM. So theoretically, a node with 24 GB RAM can be filled with 8 jobs requiring 3 GB RAM each or 6 jobs requiring 4 GB RAM each. However, not the full nominal amount of memory is really available to the jobs. Therefore, one has to reduce the memory request per job a little in order to fill a node completely. In our implementation, we reduce the memory request by 5 percent for each job, i.e. for a nominal 4 GB job, only 3.8 GB RAM are requested and for a nominal 8 GB job, we request only 7.6 GB RAM.

With our current parallelization strategy, we fit 173 hours average CPU time into an average runtime of 21 hours per exome. So we achieve a more than 8-fold acceleration of the analysis in the HPC environment in comparison to a single core computer. This is not a massive speedup for a single analysis, but our workflow is optimized rather for high throughput. When we start one exome analysis every 30 minutes, we currently reach a throughput of about 290 exomes per week. This throughput matches well with our current sequencing capacities with some additional room for the processing of external data. Note that both speed and throughput are also bounded by the total load of the HPC cluster, which we cannot completely control. Nevertheless, the workflow can be further parallelized and more analyses can be run in parallel when required in the future.

### Stability

We included a number of features in order to make the workflow run as stable as possible. The main aspect here is to treat the HPC environment with care in order to prevent system instabilities. We also have to consider that we share the cluster with other users and therefore need to follow a certain etiquette. The main measures that contribute to the stability of the workflow are:

#### Strict separation of organization and computation

The organizational work is only done in the masterscript, computations are only done in the jobscripts. Computations in the masterscript are prohibited because they put computational load on the frontend node which is vital for the HPC cluster for login, data transfer and job submission.

#### Restriction of automatic accesses to the parallel filesystem

In the event of a non-responding parallel filesystem, the workflow execution is stopped automatically. This is achieved by a lockfile mechanism (S2 Supporting Information): The masterscript sets a lockfile before every access to the parallel file system and removes it again after the access is finished. Accesses to the parallel filesystem are e.g., `ls`on a directory in the parallel filesystem, or an `mkdir`command that creates a directory in the parallel filesystem. If the parallel filesystem hangs, the access command does not return and the lockfile is not removed. The lockfile is placed in the local filesystem on the frontend node. Note that only the masterscript sets these lockfiles when accessing the parallel filesystem. The job scripts that do the computations do not set such locks. Also, the majority of the lockfiles persist only a few milliseconds (and none persist longer than 5 seconds) if the parallel filesystem is responding. When it starts, the masterscript checks whether a lockfile is present. If this is the case and the lockfile persists for 25 seconds, the masterscript exits. This way, the automated workflow is essentially shut down if the parallel filesystem is non-responding. The purpose of this measure is to prevent a build-up of queries to the parallel filesystem and thus support the system administrator’s debugging efforts. It has proven useful in practice and is not creating a bottleneck in our experience when the HPC system is stable.

#### Shut-down switch

The workflow has a built in shut-down switch that can be activated by the HPC administrators at any time. This shut-down switch is implemented as a semaphore file in the local filesystem of the frontend node (S6 Supporting Information). If it is present, the masterscript exits right at the beginning or if it is already running, at dedicated exit points. The shut-down switch is used regularly when a cluster goes into maintenance and is closed down for operation. It can also be activated in case of a system problem or when the workflow is causing or contributing to system instability. When activated or deactivated, an automatic email is sent to the workflow operators by a cronjob that monitors the presence of the semaphore file.

#### Delayed start

When new data is available for processing, the cron deamon starts one instance of the masterscript every 30 minutes. This is a measure to prevent two bottlenecks: the overloading of the scheduler with too many simultaneous job submissions and the setting of too many simultaneous lockfiles by the running masterscript instances when accessing the parallel filesystem. It is also useful to balance the resource usage of the workflow as a whole. The workflow contains jobs with higher and lower resource requirements. By starting with a delay time of 30 minutes between the instances, simultaneous submission of large jobs and a subsequent increase in queuing times is prevented.

#### No ssh to remote servers

To prevent instances of the masterscript to get stuck in non-returning ssh commands, we do not access remote servers from the masterscript. All sample-related information that has to be queried from the LIMS for running the analysis is provided in an XML configuration file that is uploaded to the cluster together with the data. The configuration file is written by another script that runs on a separate server and queries the LIMS. Similarly, the transfer of the results to the Oracle database and varbank webserver is not done by the masterscript. Instead, it writes a transfer XML file that contains the location of the result files. The transfer XML file is then picked up by a script running on another server that is doing the data transfer via scp. There is also no access to remote servers in the job scripts because the outgoing network connections of the compute nodes are reserved for the exchange of software license information only on our HPC clusters. Also, accesses to remote servers would slow down the computations considerably which is why they should be avoided in HPC workflows in general.

#### Automatic deletion of results

Space is limited on the cluster and NGS data are huge. Even larger than the input data are the temporary files that have to be stored in order to pass the data from one processing step to the next within every workflow module. Once the analysis is complete, these files are obsolete and only take up space. Therefore, these temporary files are automatically deleted after successful completion of the analysis. If the workflow is aborted because of errors, the temporary files are kept for debugging purposes. The final results from each workflow module (bam files, vcf files, statistics files, and variant tables) are directly written to a dedicated results directory. Here, the data is kept except for those files that are transferred to the database and webserver. This includes the bam files, which are the largest result files and the input fastq files. We only delete these files when the comparison of the md5 checksums [41] of the original and the transferred files shows that the transfer was complete and correct. If the fastq or bam files are needed again on the cluster for further analyses, they can be retrieved from the webserver.

### Robustness

The purpose of an automated workflow is to enable high throughput data analysis without user intervention. Therefore, the workflow implementation must be able to reliably recognize errors and react to them.

#### Several attempts of job submission

One source for workflow failure is the overload of the scheduler. If too many jobs are submitted simultaneously, the scheduler can reject a job submission. These situations are usually resolved after a short time and therefore the masterscript retries a failed job submission twice after waiting a few minutes.

#### Resubmission of failed jobs

Another source of workflow failure are job abortions. The scheduler aborts jobs that require more memory or longer walltime than requested at submission time. Therefore, the masterscript has to detect and handle aborted jobs. This is achieved via the status messages that are written by the jobscripts if they finish regularly. While a submitted job is running, the masterscript (or the child process in case of several jobs started in parallel) queries the batch system’s queue (see S1 Supporting Information, wait_for_job function). These queries are only issued every two minutes in order to not overwhelm the scheduler. Once the job has disappeared from the queue, the masterscript (or child process) checks its status messages (see S1 Supporting Infromation, check_status function). If status messages are missing, the job did not finish regularly but was aborted by the scheduler. In this case, the masterscript (or child process) automatically resubmits the job with increased memory and walltime requests. In the same way, jobs with an error status message are resubmitted. Only if the job fails again after resubmission, an error message is thrown that requires user intervention (see Fig 4).

#### Run as long as possible

Finally, the workflow should not stop if a single job fails. All modules that do not depend on the failed job are run nonetheless in order to produce the most complete result without requiring user intervention.

#### Clean exit

The masterscript tracks all submitted jobs and started child processes. When interrupted at some point during the analysis, e.g., by a kill command, it automatically kills all child processes and deletes all submitted jobs. It also completes the logfile and writes the status messages as necessary (S3 Supporting Information). It is extremely important to ensure such a clean exit. Otherwise, jobs run unsupervised and can potentially overwrite results when the workflow is restarted for the same sample again. Also, documentation of the interruption in the logfile and status table is important for debugging purposes.

### Maintainability

#### Modularity

The masterscript has a modular structure. The different types of analysis (alignment, SNP-/indel calling, CNV calling, SV calling, enrichment performance, functional annotation) can be called separately or in any combination. This simplifies debugging and extends the use of the workflow beyond the analysis of exomes. If you wanted to analyze a whole genome sequencing data set, for instance, you would simply run the alignment-, SNP- /indel calling-, SV-calling-, and functional annotation modules and skip the CNV detection- and the enrichment performance module that are suited for target enriched data only.

#### Logfile and status table

In order to ease the debugging of failed runs, a logfile is written for every sample that is analyzed. It contains all job submission calls and jobids. Detailed error messages can be looked up in the stdout/stderr output of the jobs that is directed to separate files which are identifiable via the job id. Furthermore, the masterscript writes the status (Running, Error, Finished) of every module to an sqlite table that is accessible via a webinterface (S4 Supporting Information). This table greatly helps in monitoring the automated execution of the workflow as a whole. The workflow operators can see at a glance which samples produced errors and react accordingly.

#### Configuration file

The workflow is configured for each sample with the help of an XML configuration file. In addition to the sample specific information from the LIMS, it contains all input and output paths for processing. Not hardcoding the paths in the masterscript makes it possible to change the directory structure on the cluster without changing a single line of code in the workflow scripts. Only the script that writes the XML files has to be updated in that case.

## Conclusion

We have developed a fast automated workflow for NGS data analysis by leveraging the power of HPC. It is able to process 290 exomes per week on the current IT-infrastructure. This throughput is achieved by parallelization on the one hand, and special measures to make the workflow run smoothly on the HPC infrastructure on the other hand. The resulting code is organized as a collection of modules that can be combined as necessary for different kinds of analyses. This way, the workflow can be configured to analyze exome data as well as whole genome data or any target enriched NGS data. It works for both single-read and paired-end data created in-house at the Cologne Center for Genomics (CCG) or from external sources. Furthermore, it is not limited to human sequence data but can handle any organism with a known reference genome.

The results from the workflow are accessible via the varbank webinterface where sample owners can log in, browse through the variant lists and view the alignments. They can also download result files and even raw data files, if they wish to perform additional analyses. Since its launch in October 2012, we analyzed more than 5000 exomes and uncovered the genetic background of various diseases (see e.g., [10–13]).

Although running satisfactory at the moment, the workflow is always under development. We are constantly evaluating tools released by the bioinformatics community for inclusion in the pipeline. Furthermore, the pipeline code is continuously optimized and adapted to enhance stability and speed. In this context, we will also investigate the benefits of Big Data solutions for NGS data analysis. While our approach works well currently, it is not sure it will scale with the expected growth of NGS data over the next ten years. Integration of dedicated hardware for typical tasks in NGS data analysis and adaptation of cloud computing strategies for our HPC infrastructure are two options we consider to make our workflow fit for future requirements.

Finally, we would like to emphasize the benefits of the close cooperation between the CCG and RRZK. The RRZK provides much more compute power than the CCG would have been able to establish by itself. Utilizing an already existing resource also saves operational costs, installation time, and space. And as both centers are located next door to each other, data transfer is fast via broadband network connections.

Bringing together the bioinformatics expertise and HPC expertise from both centers was key to develop the solutions presented in this paper. They may serve as an example for other high throughput data analysis projects using a similar infrastructure.

## Supporting Information

S1 Supporting Information Job Submission Function. (DOCX)

S2 Supporting Information Lockfiles for Restriction of Parallel Filesystem Access. (DOCX)

S3 Supporting Information Cleanup Function. (DOCX)

S4 Supporting Information Filling of the sqlite Status Table. (DOCX)

S5 Supporting Information Modularity. (DOCX)

S6 Supporting Information Shutdown Switch. (DOCX)
