# Supplementary material for: Leveraging the Power of High Performance Computing for Next Generation Sequencing Data Analysis: Tricks and Twists from a High Throughput Exome Workflow
Source: PLoS One. 2015 May 5;10(5):e0126321. doi: 10.1371/journal.pone.0126321 (PMC4420499; doi:10.1371/journal.pone.0126321)
Supplement: S1 Supporting Information — (DOCX) [file pone.0126321.s001.docx]

# S1 Job submission function submit_job

1 submit_job() {

2 local STATUSDIR=$1

3 local SCRIPT=$2

4 local CPUS=$3

5 local WALLTIME=$4

6 local MEM=$5

7 local ARRAY_INDICES=$6

8 local ARGS=$7

9 [ $ARRAY_INDICES == "none" ] && NJOBS=1

10 [ $ARRAY_INDICES != "none" ] && NJOBS=${ARRAY_INDICES#*-}

11 SCRIPTNAME=${SCRIPT##*/}

12 for I in 1 2; do

13 rm -f $STATUSDIR/$SCRIPTNAME*.error

14 submit $SCRIPT $CPUS $WALLTIME $MEM $ARRAY_INDICES $ARGS

15 wait_for_job

16 check_status $SCRIPTNAME $ARRAY_INDICES $NJOBS $STATUSDIR

17 if [ $FAILED -eq 1 ]; then

18 ARRAY_INDICES=$RESUBMIT_INDEX

19 MEMORYGB=${MEM%gb}

20 MEMORYMB=${MEMORYGB%mb}

21 if [ $MEMORYGB == $MEMORYMB ]; then ## gb units

22 ((MEM=$MEMORYGB+2))

23 MEM=${MEM}gb

24 else ## memory string is in mb units

25 ((MEM=$MEMORYMB+2000))

26 MEM=${MEM}mb

27 fi

28 TOTALSECONDS=`echo $WALLTIME | awk -F: \\

29 '{ print 2 * (($1 * 3600) + ($2 * 60) + $3) }'`

30 ((H=$TOTALSECONDS/3600))

31 ((M=($TOTALSECONDS%3600)/60))

32 ((S=$TOTALSECONDS%60))

33 WALLTIME=`printf "%02d:%02d:%02d" $H $M $S`

34 else

35 break

36 fi

37 done

38 }

The submit_job function wraps job submission (submit function), job monitoring (wait_for_job function) and job status check (check_status function) into one workflow. Lines 9-10 compute the number of initially submitted jobs from the $ARRAY_INDICES variable and store it in $NJOBS. This variable is used later in the check_status function. Two cycles of submit (line 14), wait_for_job (line15) and check_status (line16) are run (lines 12-37), where the second cycle submits with increased resource requests (lines 17-33). If the first submission cycle is already successful, i.e. the job finished successfully, the second cycle is skipped (lines 34-36). The $FAILED and $RESUBMIT_INDEX variables are set by the check_status function and indicate whether the job as a whole failed and which array tasks have to be resubmitted, respectively.

## submit function

The submit function encapsulates the job submission command and translates into the respective batch-system’s syntax (SLURM or TORQUE/Maui).

1 submit() {

2 local SCRIPT=$1

3 local CPUS=$2

4 local WALLTIME=$3

5 local MEM=$4

6 local ARRAYIDS=$5

7 local VARS=$6

8 local SUCCESS=0

9 VARS=`echo $VARS | awk '{gsub(/=,/,"=none,",$0); print}'`

10 STDOUTDIR=/scratch/ccg-ngs/stdout

11 SCRIPTNAME=${SCRIPT##*/}

12 VARS="$VARS,scriptname=$SCRIPTNAME"

13 if [ $HOSTNAME == "CHEOPS" ]; then

14 VARS=`echo ${VARS//,/ } | \\

15 awk 'BEGIN {RS=" "; ORS=" "};{gsub(/.+=/,""); print}'`

16 OPTIONS="-p smp -n $CPUS -t $WALLTIME --mem=$MEM \\

17 --account=ccg-ngs --mail-type=FAIL --mail-user=$EMAIL"

18 if [ $ARRAYIDS == "none" ]; then

19 STDOUT=$STDOUTDIR/$SCRIPTNAME.%j.stdouterr

20 else

21 STDOUT=$STDOUTDIR/$SCRIPTNAME.%A.stdouterr-%a

22 OPTIONS="$OPTIONS --array $ARRAYIDS"

23 fi

24 OPTIONS="$OPTIONS -o $STDOUT"

25 for I in 1 2 3; do

26 JOBID=`sbatch $OPTIONS ${SCRIPT} $VARS | cut -d " " -f 4`

27 [ ! -z $JOBID ] && SUCCESS=1 && break

28 $SLEEP 120

29 done

30 if [ $SUCCESS -eq 0 ]; then

31 echo "[`date`] ${0##*/}: Error: Cannot submit $SCRIPT job"

32 else

33 JOBID=${JOBID%%.*}

34 RUNNING_JOBS=`echo $RUNNING_JOBS+$JOBID`

35 echo "[`date`] ${0##*/}: Starting $SCRIPT [Jobid=$JOBID]"

36 fi

37 elif [ $HOSTNAME == "SuGI" ]; then

38 OPTIONS="-q default -l nodes=1:ppn=$CPUS -l walltime=$WALLTIME \\

39 –l mem=$MEM -A ccg-ngs -m a -M $EMAIL -j oe"

40 if [ $ARRAYIDS == "none" ]; then

41 STDOUT=$STDOUTDIR/$SCRIPTNAME.'${PBS_JOBID%%.*}'.stdouterr

42 else

43 STDOUT=$STDOUTDIR/$SCRIPTNAME.'${PBS_JOBID%%-*}'.stdouterr

44 OPTIONS="$OPTIONS -t $ARRAYIDS"

45 fi

46 OPTIONS="$OPTIONS -o $STDOUT"

47 for I in 1 2 3; do

48 JOBID=`qsub ${SCRIPT} $OPTIONS -v $VARS`

49 [ ! -z $JOBID ] && SUCCESS=1 && break

50 sleep 120

51 done

52 if [ $SUCCESS -eq 0 ]; then

53 echo "[`date`] ${0##*/}: Error: Cannot submit $SCRIPT job"

54 else

55 JOBID=${JOBID%%.*}

56 RUNNING_JOBS=`echo $RUNNING_JOBS+$JOBID`

57 echo "[`date`] ${0##*/}: Starting $SCRIPT [Jobid=$JOBID]"

58 fi

59 fi

60 sleep 30

61 }

The submit function takes the arguments for the jobscript as a comma-separated string of “name=value” components in the $VARS variable. Line 9 corrects the $VARS variable by including a “none” string for missing values. Line 10 specifies the directory where stdout/stderr output of the jobscript should be directed.

Line 11 cuts the path from the $SCRIPT variable and stores it in the $SCRIPTNAME variable. This variable is passed to the jobscript upon submission which uses it as the prefix for the status message files. Lines 13-36 perform the job submission in SLURM syntax on the CHEOPS cluster. Lines 14-15 split the $VARS “name=value” pairs into a BASH-like argument string of values separated by spaces. Lines 16-24 build the options string for the sbatch submission command while lines 25-29 do the actual job submission. Job submission is attempted thrice in a for-loop which breaks when a jobid has been returned from the sbatch command (line 27). The waiting time of 120 seconds (line 28) is sufficient to reach the next scheduling cycle of the batch system after a failed job submission attempt. Lines 30-31 print an error message if job submission failed. Lines 32-35 add the jobid to the masterscript’s global $RUNNING_JOBS variable that tracks all submitted jobs and print a success message. Lines 37-59 do the same procedure for the TORQUE/Maui syntax for job submission on SuGI.

## wait_for_job function

The wait_for_job function monitors the queue and waits for the jobid stored in variable $JOBID to disappear:

1 wait_for_job() {

2 if [ ! -z $JOBID ]; then

3 if [ "$HOSTNAME" == "CHEOPS" ]; then

4 CHECK_QUEUE=squeue

5 local JOBPATTERN=$JOBID

6 fi

7 if [ "$HOSTNAME" == "SuGI" ]; then

8 CHECK_QUEUE=qstat

9 local JOBPATTERN=${JOBID%%.*}

10 fi

11 while true; do

12 JOBS=`$CHECK_QUEUE`

13 if [ $? -eq 0 ]; then

14 if [ `echo $JOBS | grep $JOBPATTERN | wc -l` -eq 0 ]

15 then

16 break

17 fi

18 else

19 echo "[`date`] ${0##*/}: Error checking queue: \\

20 JOBS=$JOBS"

21 fi

22 sleep 120

23 done

24 echo "[`date`] ${0##*/}: Job $JOBID finished."

25 RUNNING_JOBS=`echo $RUNNING_JOBS | \\

26 awk -v id=$JOBID '{sub("+"id"","",$0); print}'`

27 else

28 echo "[`date`] ${0##*/}: No jobid to wait for."

29 fi

30 }

Lines 2-26 wait for the job specified via the global $JOBID variable to disappear from the queue. Lines 3-6 apply the SLURM syntax, lines 7-10 apply the TORQUE/Maui syntax. The while-loop in lines 11-23 queries the queue (line 12) every 120 seconds (line 22) and checks if the jobid has disappeared (line 14). If this is the case, the while-loop breaks (lines 15-17) and a message is printed (line 24). If the query of the queue exits with an error, an error message is printed (lines 18-20). When the jobid has disappeared from the queue, it is deleted from the masterscript’s global $RUNNING_JOBS variable (lines 25-26). If the $JOBID variable is empty, an error message is printed (lines 27-29).

## check_status function

The check_status function checks the job’s status messages in the local filesystem and collects all failed or aborted tasks for resubmission:

1 check_status() {

2 local SCRIPT=$1

3 local ARRAY_INDICES=$2

4 local NJOBS=$3

5 local STATUSDIR=$4

6 FAILED=0

7 RESUBMIT_INDEX=""

8 if [ $ARRAY_INDICES == "none" ]; then

9 RESUBMIT_INDEX="none"

10 if [ `find $STATUSDIR -name "$SCRIPT.error" | wc -l` -ne 0 ]; then

11 FAILED=1

12 rm $STATUSDIR/$SCRIPT.error

13 fi

14 if [ `find $STATUSDIR -name "$SCRIPT.finished" | wc -l` -ne 1 ]

15 then

16 FAILED=1

17 fi

18 else

19 if [ `find $STATUSDIR -name "$SCRIPT-*.error" | wc -l` -ne 0 ]; then

20 for FILE in `ls ${STATUSDIR}/$SCRIPT-*.error`; do

21 INDEX=${FILE##*-}

22 INDEX=${INDEX%%.error}

23 FAILED=1

24 RESUBMIT_INDEX=`echo $RESUBMIT_INDEX,$INDEX`

25 done

26 fi

27 if [ `find $STATUSDIR -name "$SCRIPT-*.finished" | wc -l` -ne $NJOBS ]

28 then

29 for I in $(seq $NJOBS); do

30 if [ ! -f $STATUSDIR/$SCRIPT-$I.finished ] && \\

31 [ ! -f $STATUSDIR/$SCRIPT-$I.error ]; then

32 FAILED=1

33 RESUBMIT_INDEX=`echo $RESUBMIT_INDEX,$I`

34 fi

35 done

36 fi

37 fi

38 if [ $FAILED -eq 1 ] && [ $ARRAY_INDICES != "none" ]; then

39 RESUBMIT_INDEX=${RESUBMIT_INDEX#,}

40 fi

41 }

The check_status function checks whether all $NJOBS tasks of the jobscript $SCRIPT finished successfully. For this purpose, it checks the status message files named $SCRIPTNAME.finished / $SCRIPTNAME.error (or $SCRIPTNAME-$I.finished / $SCRIPTNAME-$I.error for jobarrays, respectively) in the $STATUSDIR directory. It defines two global variables $FAILED and $RESUBMIT_INDEX (lines 6-7). Lines 8-13 do the status check for simple jobs, i.e. no jobarrays. Here, lines 10-13 check, whether an error status message is present, while lines 14-17 check whether the finished message is missing. In both cases, the global $FAILED variable is set to “1” which triggers resubmission or an error message in case of an already failed resubmission attempt. Lines 18-37 check the status messages of jobarrays. The first part, lines 19-26 again check for presence of error messages and collect the array indices that produced error messages in the $RESUBMIT_INDEX variable (lines 20-24). Lines 27-37 check for missing finished messages and add the indices of the array tasks where this is the case to the $RESUBMIT_INDEX variable (lines 29-35). Lines 38-40 delete a trailing comma from the $RESUBMIT_INDEX variable so that it can be used directly for job resubmission. The check_status function expects standardized status message files named $SCRIPTNAME.finished / $SCRIPTNAME.error (or $SCRIPTNAME-$I.finished / $SCRIPTNAME-$I.error for jobarrays, respectively).

These status message files are written to the local filesystem of the frontend node by the jobscripts using ssh:

1 ssh $HOST "touch $LOCAL_STATUSDIR/$SCRIPTNAME-$I.finished"

In the masterscript, a complete job submission and status check is then reduced to the following lines:

1 submit_job $LOCAL_STATUSDIR ${SCRDIR}/jobscript.sh 1 10:00:00 4gb 1-4 \\

2 arg1=$ARG1,arg2=$ARG2,local_statusdir=$LOCAL_STATUSDIR,host=$HOSTNAME

3 if [ $FAILED -eq 1 ]; then

4 echo "[`date`] ${0##*/}: Error: jobscript.sh failed."

5 ERRORCODE=1

6 ## other actions in case of error

7 fi

Here, lines 1-2 contain the call of the submit_job function, line 3 checks whether the job failed via the global $FAILED variable and lines 4-6 print error messages, set the $ERRORCODE variable and possibly perform other actions that are necessary after the specific job’s failure.
