# Supplementary material for: Leveraging the Power of High Performance Computing for Next Generation Sequencing Data Analysis: Tricks and Twists from a High Throughput Exome Workflow
Source: PLoS One. 2015 May 5;10(5):e0126321. doi: 10.1371/journal.pone.0126321 (PMC4420499; doi:10.1371/journal.pone.0126321)
Supplement: S2 Supporting Information — (DOCX) [file pone.0126321.s002.docx]

# S2 Lockfiles for Restriction of Parallel Filesystem Access

The lockfile mechanism to restrict automatic access to the parallel filesystem is implemented by the following code:

1 LOCKFILE=$LOCALDIR/locks/${0##*/}.lock

2 N=0

3 while [ $N -lt 5 ]; do

4 [ ! -f $LOCKFILE.* ] && break

5 N=$(( N+1 ))

6 sleep 5

7 done

8 [ $N == 5 ] && exit 0 ## File system hangs, stop script

.

.

.

9 touch $LOCKFILE.$$

10 mkdir /data/$OUTPUTDIR

11 rm -f $LOCKFILE.$$

Lines 1-8 are executed right at the beginning of the pipeline masterscript and cause an exit if a lockfile is already present and persists for 25 seconds. All accesses to the parallel filesystem are controlled by setting and removing the lockfile as listed in lines 9-11.
