# Supplementary material for: Leveraging the Power of High Performance Computing for Next Generation Sequencing Data Analysis: Tricks and Twists from a High Throughput Exome Workflow
Source: PLoS One. 2015 May 5;10(5):e0126321. doi: 10.1371/journal.pone.0126321 (PMC4420499; doi:10.1371/journal.pone.0126321)
Supplement: S3 Supporting Information — (DOCX) [file pone.0126321.s003.docx]

# S3 cleanup Function

The masterscript’s cleanup function ensures a clean exit and removes intermediate results if the pipeline finishes successfully. It is invoked whenever the masterscript exits via the trap command:

1 function cleanup(){

2 echo "[`$DATE`] ${0##*/}: Cleanup on exit: ERRORCODE=$ERRORCODE"

3 echo "RUNNING_JOBS=${RUNNING_JOBS}"

4 echo "RUNNING_PROCESSES=${RUNNING_PROCESSES}"

5 RUNNING_JOBS=${RUNNING_JOBS#+}

6 if [ $HOSTNAME == "CHEOPS" ]; then

7 DELETE_JOB=scancel

8 elif [ $HOSTNAME == "SuGI" ]; then

9 DELETE_JOB=qdel

10 fi

11 for JID in ${RUNNING_JOBS//+/ }; do

12 echo "[`$DATE`] Terminating job $JID"

13 $DELETE_JOB $JID

14 done

15 RUNNING_PROCESSES=${RUNNING_PROCESSES#+}

16 for PID in ${RUNNING_PROCESSES//+/ }; do

17 echo "[`date`] ${0##*/}: Terminating process $PID"

18 kill $PID

19 done

20 cat $STDOUTF >> $LOGFILE

21 rm $STDOUTF

22 case $ERRORCODE in

23 0)

24 rm $LOCALDIR/tmp/${SID}_${AID}*

25 touch $LOCKFILE.$$

26 cp $LOCALDIR/varpipe_stack/running/$XML_FILE $OUTDIR

27 rm -f $LOCKFILE.$$

28 statusdb $SID $AID Pipeline FINISHED update $HOSTNAME

29 mv $LOCALDIR/varpipe_stack/running/$XML_FILE \\

30 $LOCALDIR/varpipe_stack/finished

31 touch $LOCKFILE.$$

32 cp $LOGFILE $OUTDIR/${XML_FILE}.varpipe.log

33 rm -f $LOCKFILE.$$;;

34 1)

35 rm $LOCALDIR/tmp/${SID}_${AID}*

36 statusdb $SID $AID Pipeline ERROR update $HOSTNAME

37 mv $LOCALDIR/varpipe_stack/running/$XML_FILE \\

38 $LOCALDIR/varpipe_stack/error;;

39 *)

40 if [ $? -ne 0 ]; then

41 rm $LOCALDIR/tmp/${SID}_${AID}*

42 statusdb $SID $AID Pipeline ERROR update $HOSTNAME

43 mv $LOCALDIR/varpipe_stack/running/$XML_FILE \\

44 $LOCALDIR/varpipe_stack/error

45 fi;;

46 esac

47 }

48 trap cleanup EXIT SIGINT SIGTERM

The cleanup function is called whenever the pipeline exits (line 48). Lines 6-10 initialize the $DELETE_JOB variable with the respective job deletion commands in SLURM or TORQUE/Maui syntax. The function then deletes all running jobs that are stored in the $RUNNING_JOBS variable (lines 5-9). Some modules are designed as sub-pipelines which are started as a child process of the masterscript’s process on the cluster frontend. Also, some jobs are started via child processes to run them in parallel. Whenever the masterscript starts a child proccess, it stores the process id in the variable $RUNNING_PROCESSES. In lines 15-19, all those processes are killed. Every child process script contains itself a cleanup function and deletes all submitted jobs upon exit. Sub-pipelines only submit jobs and do not start other sub-pipelines, so the depth of the parent-child process tree is limited to two. Lines 20-21 update the sample logfile that contains all runs of the pipeline for the sample with all job submission- and sub-pipeline calls with their job- or process ids and all pipeline messages. Lines 22-46 contain different cleanup procedures depending on the content of the $ERRORCODE variable.

If the pipeline finished successfully ($ERRORCODE=0), lines 24-33 are executed. They delete the temporary files in the local filesystem (line 24) (the intermediate results in the parallel filesystem are deleted from within a job script). Lines 25-27 copy the sample’s configuration file to the results directory in the parallel filesystem. Line 28 calls the statusdb function that updates the sqlite pipeline status table and sets the pipeline status to “FINISHED” (see S4 Supplementary Information). Lines 29-30 move the configuration XML file from the “running” directory to the “finished” directory in the local filesystem, where it is kept for some time in case new analyses should be run. Lines 31-33 write a copy of the logfile to the results directory of the sample so it can be easily found if needed later. Lines 34-38 are executed if there was an error during pipeline execution ($ERRORCODE=1). They clean up temporary files in the local filesystem (line 35), write an error message to the sqlite status table (line 36), and move the sample’s XML configuration file to the “error” directory in the local filesystem (lines 37-38). Note that intermediate results in the parallel filesystem are kept in this case so that they are available to continue the analysis after the error and can be viewed for debugging purposes. If the $ERROCODE variable contains any other value and the exit status of the last command was different from 0, the error-case procedure is executed (lines 40-45).
