# Supplementary material for: Leveraging the Power of High Performance Computing for Next Generation Sequencing Data Analysis: Tricks and Twists from a High Throughput Exome Workflow
Source: PLoS One. 2015 May 5;10(5):e0126321. doi: 10.1371/journal.pone.0126321 (PMC4420499; doi:10.1371/journal.pone.0126321)
Supplement: S4 Supporting Information — (DOCX) [file pone.0126321.s004.docx]

# S4 Filling of the sqlite status table

The masterscript fills an sqlite status table several times during its execution (e.g., in the cleanup function described in Section 3). For this purpose, the statusdb function is used:

1 statusdb() {

2 local SID=$1

3 local AID=$2

4 local COLUMNS=$3

5 local STATUSES=$4

6 local MODE=$5

7 local HOST=$6

8 STATUSDB_LOCKFILE=/<path>/<to>/statusdb.lock

9 touch $LOCKFILE.$$

10 while [ -e $STATUSDB_LOCKFILE ]; do

11 sleep 3

12 done

13 touch $STATUSDB_LOCKFILE

14 rm -f $LOCKFILE.$$

15 SQLITE=/usr/bin/sqlite3

16 STATUSDB=/<path>/<to>/status.db

17 TIMESTAMP=`date +%s`

18 if [ $MODE == "insert" ]; then

19 touch $LOCKFILE.$$

20 echo "insert or replace into analysis_status values \\

21 (('$AID','$SID','','','','','','','','','','','','','RUNNING', \\

22 '$TIMESTAMP','$HOST');" | $SQLITE $STATUSDB

23 rm -f $LOCKFILE.$$

24 fi

25 if [ $MODE == "update" ]; then

26 for COL in ${COLUMNS//+/ }; do

27 STATUS=${STATUSES%%+*}

28 STATUSES=${STATUSES#*+}

29 touch $LOCKFILE.$$

30 echo "update analysis_status \\

31 set $COL=\"$STATUS\",Time=\"$TIMESTAMP\" where \\

32 AID=\"$AID\";" | $SQLITE $STATUSDB

33 rm -f $LOCKFILE.$$

34 done

35 fi

36 touch $LOCKFILE.$$

37 rm -f $STATUSDB_LOCKFILE

38 rm -f $LOCKFILE.$$

39 }

The statusdb function can be called in two modes, “insert” and “update” (line 6). It uses a statusdb-lockfile in the parallel filesystem (line 8) to prevent simultaneous access to the sqlite table by different pipeline processes, which would result in an error. At the start, it checks whether the lockfile is present and waits until it disappears (lines 10-12). Then it sets the lockfile itself before continuing (line 13). Note that like all accesses to the parallel filesystem, the checking and setting of the lockfile are surrounded by the setting and removal of the masterscript’s lockfile (lines 9 and 14). This lockfile mechanism is also employed for the access to the status table, which also resides in the parallel filesystem (lines 19, 23, 29, and 33). Lines 15 and 16 define the sqlite3 command and the location of the status table file. Every update of the table is documented by a timestamp that is defined in line 17. Lines 18-24 add a new row to the table and initialize the columns to a fixed set of values. Lines 25-35 update the columns specified in $COLUMNS of an existing row of the table with the values contained in $STATUSES.

For this purpose, the script loops over the “+” separated string of column names in $COLUMNS (line 26), retrieves the corresponding value from the “+” separated $STATUSES string (lines 27-28) and performs the table update (lines 30-32). Finally, it removes the statusdb-lockfile (lines 36-38) to free the table for other accesses.

Note that the parallel filesystem which we use for the pipeline is mounted on both HPC-clusters, CHEOPS and SuGI. This way, all input fastq-files, final result files, and the status table are accessible from both systems and we can run in parallel or switch between systems without copying or synchronizing data.
