# Supplementary material for: Leveraging the Power of High Performance Computing for Next Generation Sequencing Data Analysis: Tricks and Twists from a High Throughput Exome Workflow
Source: PLoS One. 2015 May 5;10(5):e0126321. doi: 10.1371/journal.pone.0126321 (PMC4420499; doi:10.1371/journal.pone.0126321)
Supplement: S5 Supporting Information — (DOCX) [file pone.0126321.s005.docx]

# S5 Modularity

The masterscript has a modular structure. The analysis is split into several modules that can be executed in any combination. Which modules are to be run is selected via a command line option, e.g. the following call runs modules 1,2,3,4, and 5:

./varpipe.sh <Configuration_XML_File>.xml 1,2,3,4,5

In the masterscript, the modularity is implemented by the following lines of code:

1 XML=$1 # xml file of the sample to process

2 TASKS=$2 # list of modules to execute

3 NTASKS=13 # total number of modules in the pipeline

4 for T in $(seq 1 1 $NTASKS); do # initialize task array

5 TASKARRAY[$T]=0

6 done

7 for T in ${TASKS//,/ }; do # loop through command line modules list

8 if [[ ${T} == *[a-zA-Z]* ]]; then

9 echo "[`date`] ${0##*/}: Error: Unknown task: $T"

10 exit 1

11 fi

12 if [ ${T} -gt $NTASKS ]; then

13 echo "[`date`] ${0##*/}: Error: Unknown task: $T"

14 exit 1

15 fi

16 TASKARRAY[${T}]=1

17 done

.

.

.

18 if [ ${TASKARRAY[1]} -eq 1 ]; then

19 ## Module 1 (alignment) is selected

20 ## Execute alignment

21 fi

.

.

.

22 if [ ${TASKARRAY[1]} -eq 1 ]; then

23 ## Check module status after completion

24 fi

Lines 1-2 retrieve the input arguments. Line 3 defines the number of pipeline modules that is used later in a plausibility-check of the content of the $TASKS list. In order to easily look up whether a specific module is selected for execution, we use an array that contains a “1” if the module is selected and a “0” else. This array is initialized with “0” values in lines 4-6. Lines 7-17 loop over the values of the comma-seperated $TASKS list and check it for evident typos (lines 8-11 check whether letters are contained, lines 12-15 check whether the task number is valid). If no typos are found, the array-value of the respective module is set to “1” (line 16). Execution of a specific module is then triggered by the array’s value (lines 18-21). Later in the masterscript, the status of every module that was executed during the run is checked as shown in lines 22-24.
