# Supplementary material for: Leveraging the Power of High Performance Computing for Next Generation Sequencing Data Analysis: Tricks and Twists from a High Throughput Exome Workflow
Source: PLoS One. 2015 May 5;10(5):e0126321. doi: 10.1371/journal.pone.0126321 (PMC4420499; doi:10.1371/journal.pone.0126321)
Supplement: S6 Supporting Information — (DOCX) [file pone.0126321.s006.docx]

# S6 Pipeline shutdown switch

The pipeline shutdown switch is just a semaphore file in the local filesystem. The following line of code in the beginning of the masterscript stops the pipeline, if the file is present:

[ -f /<local>/<path>/<to>/ccg.off ] && exit 0
